# Facultative Endosymbiont Serratia symbiotica Inhibits the Apterization of Pea Aphid To Enhance Its Spread

**DOI:** 10.1128/spectrum.04066-22

**Published:** 2022-11-29

**Authors:** Zhi-Wei Kang, Meng Zhang, He-He Cao, Shan-Shan Guo, Fang-Hua Liu, Tong-Xian Liu

**Affiliations:** a School of Life Science, Institutes of Life Science and Green Development, Hebei University, Baoding, Hebei, China; b State Key Laboratory of Crop Stress Biology for Arid Areas, Northwest A&F University, Yangling, Shaanxi, China; c Key Laboratory of Integrated Crop Pest Management of Shandong Province, College of Plant Health and Medicine, Qingdao Agricultural University, Qingdao, Shandong, China; d Institute of Entomology, Guizhou University, Guiyang, Guizhou, China; Chinese Academy of Sciences

**Keywords:** life history, symbiosis, trade-off, wing differentiation

## Abstract

Aphids display wing polyphenism, and the mother can produce a wingless morph for reproduction and a winged morph for dispersal. It is believed that the wingless morph is an adaptive status under favorable conditions and is determined prenatally. In this study, we have found that winged nymphs of the pea aphid, Acyrthosiphon pisum, can change from winged to wingless during normal development. Our results showed that winged nymphs could become the wingless morph by apterization in response to changes from stressful to favorable conditions. The acquired wingless aphids had higher fecundity than the winged morph. However, this process of regression from winged to wingless morph was inhibited by Serratia symbiotica. The existence of the symbiont did not affect the body mass and fecundity of adult aphids, but it increased the body weight of nymphs and temporally increased the quantity of a primary symbiont, Buchnera aphidicola. Our results showed that despite temporal improvement of living conditions causing the induction of apterization of winged nymphs, the inhibition effect of S. symbiotica on this process was activated simultaneously. This finding, for the first time, reveals that the wingless morph can be changed postnatally, which explains a novel regulating mechanism of wing polyphenism driven by external abiotic stimuli and internal biotic regulation together in aphids.

**IMPORTANCE** Wing polyphenism is an important adaptative response to environmental changes for aphids. Endosymbionts are widespread in aphids and also confer the ability to withstand unfavorable conditions. However, little is known about whether endosymbionts are involved in the wing polyphenism. In this study, we report a new finding that winged nymphs of the pea aphid could turn into adults without wings or wing-related structures through apterization when winged nymphs escaped from stressful to favorable environments. Further analysis revealed that the facultative symbiont S. symbiotica could prevent the temporal determination of the host in wing suppression by inhibiting apterization, to enhance its spread. Our findings provide a novel angle to understanding the wing polyphenism regulation of aphids.

## OBSERVATION

Most aphids show wing polyphenism, in which one genotype can produce progeny with different wing morphs in responding to various biotic and abiotic conditions, including host plant quality, population density, temperature, photoperiod, alarm pheromones, and interactions with predators, parasites, mutualists, entomopathogens, and endosymbionts (1). The wingless morph of aphids, which shows higher fecundity than winged aphids, is the adaptive status in the asexual phase under favorable conditions. Previous research shows that wing polyphenism in the pea aphid, Acyrthosiphon pisum, is determined prenatally (2); the wingless nymphs maintain the wingless status for life, and there is no evidence as to whether winged nymphs can revert to wingless adults.

Animals routinely engage in symbiotic associations with microorganisms that modulate host nutrition, immunity, and defense responses (3). Insects and other terrestrial arthropods frequently maintain infections of heritable bacterial symbionts that provide fitness benefits (4). When insects bump against biotic or abiotic pressures suddenly, endosymbionts help hosts get through or overcome adversities, which ensures that symbionts will not lose their shelter, nutrition supplying, and opportunities for further spreading. Under natural selection, symbionts extend the ecological niche of hosts and reshape communities that their hosts belong to (5). Besides facing selective pressure, the weakening or disappearance of a source of selection for an animal is also one of the most commonly encountered scenarios in the natural environment (6). Temporary weakening or disappearance of pressures for a symbiont’s hosts is especially common. Unfortunately, we know little about how symbionts affect their hosts in this situation.

**FIG 1:**
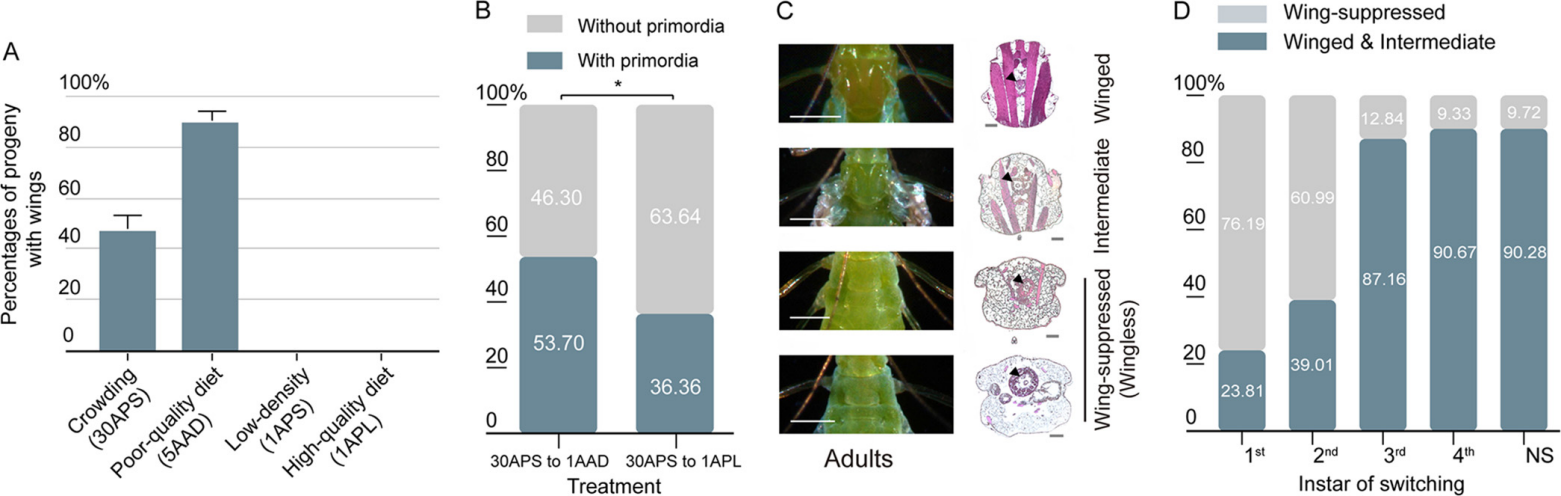
Time window of apterization of winged nymphs of A. pisum. (A) The proportions of winged progeny of pea aphids produced by wingless adults under different conditions were 47.8% ± 5.3% under a crowding condition (30APS) (*n *= 3) and 90.6% ± 3.7% on a poor-quality (artificial) diet (5AAD) (*n *= 3). The proportions of wingless progeny for mothers raised under favorable conditions (1APS and 1APL) were 100% (*n *= 3 and 3). Mean values and standard errors are shown. (B) Percentages of different wing morphs of adults that were switched from a poor-quality diet to a high-quality diet in different nymphal instars (Z = −11.4591; *P < *0.0001; *n *= 465). (C) Wing structures were suppressed when apterization occurred in A. pisum. The white and black scale bars are 500 μm and 100 μm, respectively. (D) Percentages of different wing morphs of adults that were switched from a poor-quality diet to a high-quality diet in different nymphal instars (Z = −11.4591; *P < *0.0001; *n *= 465).

Poor nutrition and high density are considered to be important factors for inducing winged morphs in aphids (2, 7). In our experiments, we found that aphids reared on a poor-quality diet (1AAD, one aphid on an artificial diet) showed lower rates of reproduction than aphids on a high-quality diet (1APL, one aphid on a plant leaf disc) (Fig. S1 in the supplemental material). Exposing wingless pea aphid mothers to crowded conditions (30APS, 30 adult aphids on one plant seedling) or a poor-quality diet (5AAD, 5 adult aphids on the artificial diet) increased the proportion of winged progeny compared with that of mothers under low-density conditions (1APS, one aphid on one plant seedling) or on a high-quality diet (1APL) (Fig. 1A). When the neonate nymphs produced by wingless females under crowded conditions (30APS) were chosen at random and reared on the poor-quality diet (1AAD) or the high-quality diet (1APL), more third instar nymphs (53.7%) became winged nymphs with wing primordia than wingless nymphs (46.3%) when they were on the poor-quality diet, but more developed to wingless nymphs (63.64%) than winged nymphs (36.36%) when they were reared on the high-quality diet (Fig. 1B). Furthermore, we found that an early switch (first and second instars) from the poor-quality diet (1AAD) to the high-quality diet (1APL) induced greater apterization, resulting in >60% wingless aphids, than a late switch (third and fourth instars), resulting in 9.33 to 12.84% wingless aphids (Fig. 1C and D; Fig. S2). Winged and wingless aphids are expected to exhibit a trade-off between dispersal and reproduction (8). Our results showed that the pea aphids showing suppressed or intermediate wings exhibited higher rates of reproduction (similar to that of wingless aphids) than winged adults (Fig. S3). Thus, we concluded that the postnatal induction of wing suppression represented a more finely tuned strategy for the aphids, allowing responses to some sudden environmental changes, which served as a complement to the prenatal wing determination. These results demonstrated that the decision to keep or discard wings could be made at the first two nymphal instars of the aphids, potentially providing them with more flexibility to develop into a morph that would be best adapted to their immediate environments.

**FIG 2:**
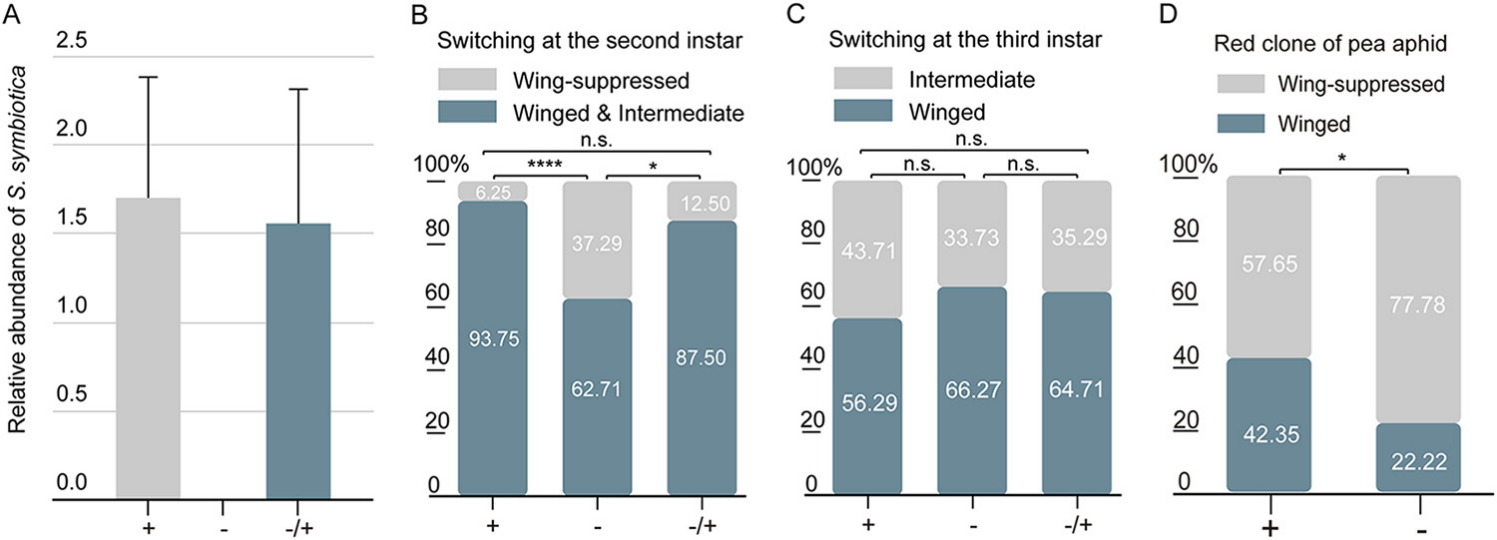
S. symbiotica inhibits apterization of winged nymphs of A. pisum. (A) Relative abundances of Serratia symbiotica in three aphid lines (*n *= 3). Mean values and standard errors are shown. +, naturally infected line; −, cured line; −/+, cured and reinfected line. (B and C) Percentages of different wing morphs of the naturally infected, cured, and reinfected aphid lines following switching of conditions from a poor-quality diet to a high-quality diet in the second (B) and third (C) instars. Second instar: naturally infected line versus cured line (chi-square = 14.1063; *P < *0.0001; contingency coefficient = 0.3427; *n *= 107); naturally infected line versus reinfected line (chi-square = 0.9393; *P = *0.3324; contingency coefficient = 0.1077; *n *= 80); cured line versus reinfected line (chi-square = 6.2466; *P = *0.0124; contingency coefficient = 0.2534; *n *= 91). Third instar: naturally infected line versus cured line (chi-square = 2.2924; *P = *0.13; contingency coefficient = 0.0953; *n *= 250); naturally infected line versus reinfected line (chi-square = 1.4129; *P = *0.2346; contingency coefficient = 0.0773; *n *= 235); cured line versus reinfected lineage (chi-square = 0.0402; *P = *0.841; contingency coefficient = 0.0163; *n *= 151). +, naturally infected line; −, cured line; −/+, cured and reinfected line; *, significant difference at *P < *0.05; ****, *P *< 0.0001; n.s., not significantly different. (D) Percentages of different wing morphs of the cured and naturally infected red aphid line after they were switched from 1A 0.3×AD (one aphid on a poor-quality diet that contained 30% of the previously used artificial diet) to 1APL (chi-square = 5.9662; *P = *0.0146; contingency coefficient = 0.1937; *n *= 157). +, naturally infected line; −, cured line; *, mean values are different at *P < *0.05 (chi-square test). Numbers in histograms are sample sizes.

Aphids harbor an obligate endosymbiont, Buchnera aphidicola, which supplies essential amino acids that aphids cannot synthesize, and various facultative endosymbiont bacteria, which provide aphids with many new traits for coping with stressful conditions (1, 7, 9). In this study, only the primary endosymbiont, B. aphidicola, and a single facultative symbiont, Serratia symbiotica, were detected in the green pea aphid clone that we used (Fig. S4). Because loss of the primary symbiont can lead to infertility or severe dysplasia in aphids (1), we used selective antibiotic treatments, which had no impact on the relative abundance of B. aphidicola, to generate a “cured” aphid line (−) that was genetically identical to the naturally infected line (+) and differed only in the absence of S. symbiotica (Fig. 2A; Fig. S5). To verify the impact of S. symbiotica on apterization, we also created a reinfected aphid line (−/+) by injecting the hemolymph of naturally infected nymphs into a cured aphid line (−) with the same genetic background. We found that the cured aphid line showed more apterous aphids than the naturally infected and reinfected lines when the aphids were transferred from the poor-quality diet (1AAD) to the high-quality diet (1APL) in the second instar, but not in the third instar (Fig. 2B and C). Meanwhile, when the nymphs produced by adults reared under the crowded conditions (30APS) were maintained on a poor-quality diet (1AAD), the percentages of winged morphs of the naturally infected S. symbiotica and cured lines were similar (Fig. S6). To elucidate whether the inhibition of apterization by S. symbiotica was conserved among the different pea aphid genotypes, we used a red clone of the pea aphid that also only harbors B. aphidicola and S. symbiotica. We found that the cured aphids also showed stronger apterization than the naturally infected line (Fig. 2D). This result further demonstrated that the effect of S. symbiotica on polyphenic wing development could be generalized across different pea aphid genotypes.

However, the body weights of the naturally infected and reinfected lines were significantly heavier than the body weight of the uninfected line at the second instar, which was the critical time stage of apterization (Fig. S7A). We did not find significant differences in fecundity or offspring size (Fig. S7B and C), but the development time of the nymph was reduced in the reinfected aphids (Fig. S7D). The relative abundances of B. aphidicola in the cured line were significantly lower than in the other two lines in first, second, and third instar nymphs (Fig. S7E). It has been reported that the primary endosymbiont, B. aphidicola, is necessary for normal growth and reproduction in A. pisum (7, 10). Our results suggested that there was a small fitness advantage from S. symbiotica infection, which seems to be oppositive to our finding of the inhibition of apterization by S. symbiotica infection. Thus, the potential mechanism of the effect of S. symbiotica on the wing polyphenism in pea aphids still needs further investigation.

In conclusion, our findings enrich the understanding of wing polyphenism in pea aphids. For aphids, reversion to the apterous morph could promote responsiveness to temporal environmental changes, including population density and habitat quality. However, the influence of S. symbiotica on apterization could enhance the spread of this symbiont for its own benefit. Our findings are the first evidence that winged nymphs become wingless adults during normal development in aphids and the first to show that the facultative symbiont S. symbiotica also has influence on the polyphenism of the host as transitions occur between favorable and stressful environmental conditions. Because temporal releasing of selective pressure and facultative endosymbionts are widespread, the combined or joint influence of these abiotic and biotic factors on forming certain phenotypes of host animals is likely more important than has been thought.

## References

[1] van Emden HF, Harrington R. 2007. Aphids as crop pests. CABI Press, Wallingford, UK.

[2] Müller CB, Williams IS, Hardie J. 2001. The role of nutrition, crowding and interspecific interactions in the development of winged aphids. Ecol Entomol 26:330–340. doi:10.1046/j.1365-2311.2001.00321.x.

[3] Zilber-Rosenberg I, Rosenberg E. 2008. Role of microorganisms in the evolution of animals and plants: the hologenome theory of evolution. FEMS Microbiol Rev 32:723–735. doi:10.1111/j.1574-6976.2008.00123.x. 18549407

[4] Moran NA, McCutcheon JP, Nakabachi A. 2008. Genomics and evolution of heritable bacterial symbionts. Annu Rev Genet 42:165–190. doi:10.1146/annurev.genet.41.110306.130119. 18983256

[5] Ferrari J, Vavre F. 2011. Bacterial symbionts in insects or the story of communities affecting communities. Philos Trans R Soc Lond B Biol Sci 366:1389–1400. doi:10.1098/rstb.2010.0226. 21444313 PMC3081568

[6] Lahti DC, Johnson NA, Ajie BC, Otto SP, Hendry AP, Blumstein DT, Coss RG, Donohue K, Foster SA. 2009. Relaxed selection in the wild. Trends Ecol Evol 24:487–496. doi:10.1016/j.tree.2009.03.010. 19500875

[7] Shigenobu S, Watanabe H, Hattori M, Sakaki Y, Ishikawa H. 2000. Genome sequence of the endocellular bacterial symbiont of aphids *Buchnera* sp. APS. Nature 407:81–86. doi:10.1038/35024074. 10993077

[8] Mackay PA, Wellington W. 1975. A comparison of the reproductive patterns of apterous and alate virginoparous *Acyrthosiphon pisum* (Homoptera: Aphididae). Can Entomol 107:1161–1166. doi:10.4039/Ent1071161-11.

[9] Oliver KM, Degnan PH, Burke GR, Moran NA. 2010. Facultative symbionts in aphids and the horizontal transfer of ecologically important traits. Annu Rev Entomol 55:247–266. doi:10.1146/annurev-ento-112408-085305. 19728837

[10] Wilson ACC, Ashton PD, Calevro F, Charles H, Colella S, Febvay G, Jander G, Kushlan PF, Macdonald SJ, Schwartz JF, Thomas GH, Douglas AE. 2010. Genomic insight into the amino acid relations of the pea aphid, *Acyrthosiphon pisum*, with its symbiotic bacterium *Buchnera aphidicola*. Insect Mol Biol 19:249–258. doi:10.1111/j.1365-2583.2009.00942.x. 20482655

