# Supplementary material for: Facultative Endosymbiont Serratia symbiotica Inhibits the Apterization of Pea Aphid To Enhance Its Spread
Source: Microbiol Spectr. 2022 Nov 29;10(6):e04066-22. doi: 10.1128/spectrum.04066-22 (PMC9769995; doi:10.1128/spectrum.04066-22)
Supplement: Supplemental file 1 — Supplemental material. Download spectrum.04066-22-s0001.pdf, PDF file, 0.7 MB [file spectrum.04066-22-s0001.pdf]

Supplemental Information:

A facultative endosymbiont *Serratia symbiotica* inhibits the apterization of pea aphid to enhance its spread

Zhi-Wei Kang, Meng Zhang, He-He Cao, Shan-Shan Guo, Fang-Hua Liu*, and Tong-Xian Liu*

*Correspondence author.

E-mail address: liufanghua5@163.com (F.H. Liu), tx.liu@gzu.edu.cn (T.X. Liu).

Supplement File:

MATERIAL AND METHODS

FIGURES: 7

REFERENCES: 11

MATERIAL AND METHODS

Aphid stocks and maintenance. The two clones of *A. pisum* used in this study were collected from Yunnan Province (green morph) and Gansu Province (red morph), China. From each clone, one parthenogenetic female was selected at random to establish a clonal experimental line. Prior to experimentation, each of the *A. pisum* clones was maintained parthenogenetically for a minimum of 10 generations under long-day photoperiod in an insectary (14L : 10D, 18°C). The green clone was used as primary testing material, and the red clone was used to validate the results from the green clone.

Suitability evaluation of different diet conditions. First instar *A. pisum* nymphs were produced by wingless asexual females under three low-density conditions: (A) a single adult aphid was reared on a single plant seedling (1APS), as the most favorable condition; (B) neonate nymphs (born within 24 h) were reared on a plant leaf disc (1APL), as the high-quality diet condition; and (C) neonate nymphs were reared on an artificial diet (1AAD), as the least favorable condition compared with 1APL. The artificial diet was sandwiched between two layers of Parafilm® in a plastic cage with screen (100 mesh) at the top for ventilation. Leaf discs were individually placed on a thin layer (3-4 mm in thickness) water-agar gel (0.9% agar) in the bottom of a rearing cage (1 cm diameter) to maintain consistent leaf turgor. Small cages (1 cm diameter) were prepared for rearing single aphids. Artificial diet and leaf disc were changed every 48 h. The survival and fecundity of the adult aphids were recorded daily until death.

Winged aphid induction and determination of wing morph. We induced wing formation via crowding, by placing 30 wingless adult aphids on a 2 cm tall broad bean seedling (30APS) for 24 h. The number of nymphs produced on the bean seedling with and without wing primordia was recorded. We also induced wing formation by placing five wingless adults on the poor-quality artificial diet (5AAD) in a cage (3 cm diameter), and neonate nymphs were collected after 24 h and were then reared individually on the artificial diet in a small cage. To obtain wingless aphids, adults were reared under low-density (1APS) or high-quality diet (1APL) conditions. The neonate nymphs were transferred to bean seedlings or a new leaf disc 24 h later, and then they were reared individually.

For testing effects of postnatal treatments on wing polyphenism in *A. pisum*. A cohort of neonate nymphs from wingless females were collected over a period of 24 hours and transferred to a low-quality diet (1AAD) or a high-quality diet (1APS) conditions. Third instars were inspected using a dissecting microscope (×60 magnification) (JSZ6, Nanjing Jiangnan Novel Optics, Jiangsu, China). Those exhibiting wing primordia were collected and maintained on broad bean seedlings individually until the nymphs developed to fourth instar, and the ones showing wing primordia was recorded.

To determine how and when postnatal treatments affected wing differentiation, the poor-quality diet (5AAD) condition, which resulted in >90% winged aphids, was used to induce winged nymphs. Twenty percent of first instar nymphs were individually transferred to leaf discs (1APL), while the remaining 80% were reared solely on the poor-quality (artificial) diet (1AAD). When the nymphs feeding on the artificial diet developed to second instar, another 20% were transferred to the 1APL condition, the remaining 60% were still on the

artificial diet. This process continued until all nymphs matured. The wing morphs of adult aphids were detected, recorded, and photographed using a stereoscopic microscope (Discovery.V12; Carl Zeiss, Gottingen, Germany) combined with an AxioCam MRc 5 digital microscope camera and AxioVision image analysis software (Carl Zeiss, Gottingen, Germany).

Histological examination of adult aphids with different wing morphs. We prepared resin sections of adult aphids to compare flight muscle development among winged morphs as described previously with modifications (1). The aphids were fixed in 2.5% glutaric dialdehyde overnight and dehydrated in increasing concentrations of ethanol (50, 70, 80, 90 and 100%), and after being embedded in 1% osmic acid, further dehydrated using 100% acetone twice. They were then transferred into 25, 50 and 75% Epon812 epoxy resin for 2, 5 and 12 h, respectively, before being embedded in 100% Epon812 epoxy resin for 48 h. They were then held at 30°C for 24 h, and then dried at 60°C for 24 h. Five-micrometer thick slices were obtained with a microtome (Leica EM UC7, Leica Corporate, Solms, Germany) and stained with hematoxylin and eosin. The aphid tissues were observed through an Eclipse 80i fluorescent microscope (×100 magnification) (Nikon, Tokyo, Japan), and images were captured using a DS-Ri1 digital camera (Nikon, Tokyo, Japan) which was fixed on the microscope and linked to a computer. These images were analyzed by Nikon NIS Elements software (Nikon, Tokyo, Japan). The esophageal valve, an invagination of the foregut into the stomach that loops twice in thoracic cross-sections, was chosen as a marker to ensure that the sections selected from different aphids were taken from the same position (2).

Endosymbiont detection and microinjection procedure. For detection of facultative endosymbiotic bacteria in the aphid, a series of specific primers were used in diagnostic PCR analysis (unique primers of eight endosymbiont were referenced from reference) (3). Meanwhile, we also screened for unexpected symbionts using universal 16s primers (16SA1: AGAGTTTGATCMTGGCTCAG and 16SB1: TACGGYTACCTTGTTACGACTT) and following amplification conditions: 94°C for 2 min; followed by 30 cycles consisting of 94°C for 1 min, 50°C for 1 min, and 70°C for 2 min. DNA was extracted from the adult aphids with the Wizard® Genomic DNA Purification Kit (Promega, Madison, USA). PCR was carried out using *TaKaRa Taq*™ (TaKaRa, Dalian, Liaoning, China). PCR amplification products were extracted from 1% agarose gel and purified using a gel extraction kit (Axygen, Hangzhou, Zhejiang, China). The product was cloned using pGEM-T Easy Vector System (Promega, Madison, USA) and *E. coli* Competent Cells (TaKaRa, Dalian, Liaoning, China). 275 clones of 16s primer products from the primary experimental clonal line were selected randomly and sequenced (Invitrogen, Shanghai, China). Sequences were compared against nr/nt database using BLAST.

To create an aphid line with the same genotype but cured of *S. symbiotica*, 400 nl of 10 mg/ml of the antibiotic ampicillin was injected into the haemolymph of third instar aphids using an MM33 links microinjector (Märzhäuser Wetzlar, Germany) (4-7). The progeny was collected between 36 and 72 h after the final molt. After diagnostic PCR for progeny, an individual female aphid without *S. symbiotica* was used to establish a clonal experimental line on broad bean seedlings for at least 30 generations before used in subsequent

treatments.

Establishment of the re-infected aphid line. Twenty nanoliters of haemolymph from the third instar nymphs of the naturally *S. symbiotica*-infected aphid line were injected into the third instar nymphs of the cured line obtained from the antibiotic treatment (4, 5). The offspring produced 8-16 days after injection were collected and tested with PCR using *S. symbiotica* primers *16SrDNA* (16SA1: AGAGTTTGATCMTGGCTCAG and PASScmp: GCAATGTCTTATTAACACAT). One of those successful re-infected clones was chosen at random and allowed to multiply for subsequent experiments. The line was reared on broad bean plants for at least 10 generations before conducting the assays described below.

We conducted real-time quantitative PCR (qPCR) to compare *S. symbiotica* titers in naturally-infected, ampicillin-cured, and artificially re-infected aphids. qPCR was performed using an iCycler iQ5 Real-Time PCR Detection System (Bio-Rad, Hercules, CA, USA) and the SYBR® *Premix Ex Taq*™ II kit (Tli RNaseH Plus; TaKaRa, Dalian, China). Because the second instar is the critical stage for apterization, 30 second instar nymphs from each line were randomly collected every week for three consecutive weeks. They were frozen in liquid nitrogen, and stored at -80°C. Template DNAs from the three lines were extracted for qPCR performed under the following reaction conditions: at 95°C for 5 min; followed by 40 cycles of at 94°C for 30 s, 57°C for 30 s, and 72°C for 1 min. A melting curve analysis was performed to confirm the identity and specificity of amplified products. PCR diagnostic primers *16SrDNA* (16SA1 AGAGTTTGATCMTGGCTCAG and PASScmp GCAATGTCTTATTAACACAT) were selected to calculate the relative amount of *S. symbiotica* (2), and *EF1-alpha* (ApEF1alpha 107F CTGATTGTGCCGTGCTTATTG and ApEF1alpha 246R TATGGTGGTTCAGTAGAGTCC) from the aphid was used as a reference gene (3). The raw data of quantitative PCR were exported from IQ5-software (BioRad, Hercules, CA, USA), and the amplification efficiency and cycle threshold (Ct) value of each individual PCR reaction were determined using LinRegPCR version 12.18 (8, 9). The relative amount was calculated using the Pfaffl method (10).

Analyzing the densities of primary endosymbiont, *B. aphidicola*, in three aphid lines. The relative quantities of *B. aphidicola* in each instar of the naturally-infected, ampicillin-cured, and artificially re-infected aphid lines were measured by the same qPCR methods used for testing *S. symbiotica*. PCR diagnostic primers 16SrDNA (Buch16S1F: GAGCTTGCTCTCTTTGTCGGCAA and Buch16S1R: CTTCTGCGGGTAACGTCACGAA) were selected to calculate the relative amount of *B. aphidicola* (3), using *EF1-alpha* from the aphid as a reference gene (4).

Fitness tests for naturally-infected, cured and re-infected lines. We compared three fitness parameters to test effects of *S. symbiotica* induced changes in apterization: fresh body weight, fecundity and development time of nymphs in the three aforementioned lines. Neonate nymphs produced by the adults under low-density (1APS) conditions were transferred to the high-quality diet (1APL). Neonate aphids were counted daily for 10 days and development time was assessed until final molt. The fresh body weight of the nymphs at each instar was assessed using an analytical balance at a precision of 0.01 mg (Sartorius Weighing Technology GmbH, Goettingen, Germany). Data from the aphids that

died before the final molt were excluded from the data analysis.

Effects of *S. symbiotica* infection on wing morph production. Briefly, 30 wingless adult aphids from naturally symbiont-infected or cured lines were kept under the crowded (30APS) conditions. Neonate nymphs were removed 24 h later, and transferred individually to a poor-quality artificial diet (1AAD) under low-density on bean plant seedlings (1APS) until they developed to third instar. Wing morphs of the nymphs were visually scored based on the presence of wing primordia.

Late second or early third instar nymphs produced by the wingless females of naturally-symbiont infected, cured and re-infected lines on the poor-quality diets (5AAD) were transferred to the high-quality diets (1APL). Nymphs without primordia were discarded when they developed to third instar. Wing morphs were observed and recorded after the final molt.

Experiments using a red *A. pisum* clone. Presence of facultative endosymbionts in a red clone of *A. pisum* from Gansu Province in northwestern China were tested using a series of special primers (3). We repeated two experiments, including postnatal apterization and comparing the naturally-infected *S. symbiotica* red clone and its cured, symbiont-free counterpart with the same genotype to validate results that obtained with the green clone of *A. pisum*. Neonate nymphs produced within 24 hours by wingless red adults on poor-quality diet conditions were selected and reared individually on artificial diets. Newly molted second instars were transferred to plant discs (1APL). Nymphs with primordia were selected after the next molt and maintained on the plant discs until they developed to the fourth instar. The number of the fourth instar nymphs which still had wing primordia was recorded.

Concurrently, a cured line of the red clone of *A. pisum* was established using the same method as for the green clone and maintained along with uncured nymphs under 5AAD condition. Preliminary experiments showed that the performance of the red clones on the previously used artificial diet was similar to the aphids that were reared on plants, and the apterization was weak after changing conditions (11). To generate a low-quality artificial diet for poorer performance and stronger apterization of aphid nymphs than those on the previously used artificial diet, we diluted the diet with 70% ddH_2O so that the new artificial diet contained only 30% (0.3×AD) of the original diet. Then, individual second instar nymph was switched from low quality artificial diet condition (0.3×AD) to high quality diet condition (1APL). The third nymphs that were lack of wing primordia were discarded. The cured and uncured lines were compared for presence or absence of wings at adult stage.

REFERENCEs

1. Kodama H, Yamasaki A, Nose M, Niida S, Ohgame Y, Abe M, Kumegawa M, Suda T. 1991. Congenital osteoclast deficiency in osteopetrotic (op/op) mice is cured by injections of macrophage colony-stimulating factor. J Exp Med 173:269-272.
2. Ishikawa A, Miura T. 2009. Differential regulations of wing and ovarian development and heterochronic changes of embryogenesis between morphs in wing polyphenism of the vetch aphid. Evol Dev 11:680-688.
3. Tsuchida T, Koga R, Shibao H, Matsumoto T, Fukatsu T. 2002. Diversity and

geographic distribution of secondary endosymbiotic bacteria in natural populations of the pea aphid, *Acyrthosiphon pisum*. Mol Ecol 11:2123-2135.

4. Oliver KM, Russell JA, Moran NA, Hunter MS. 2003. Facultative bacterial symbionts in aphids confer resistance to parasitic wasps. Proc Natl Acad Sci USA 100:1803-1807.
5. Oliver KM, Moran NA, Hunter MS. 2005. Variation in resistance to parasitism in aphids is due to symbionts not host genotype. Proc Natl Acad Sci USA 102:12795-12800.
6. Koga R, Tsuchida T, Sakurai M, Fukatsu T. 2007. Selective elimination of aphid endosymbionts: effects of antibiotic dose and host genotype, and fitness consequences. FEMS Microbiol Ecol 60:229-239.
7. Tsuchida T, Koga R, Horikawa M, Tsunoda T, Maoka T, Matsumoto S, Simon JC, Fukatsu T. 2010. Symbiotic bacterium modifies aphid body color. Science 330:1102-1104.
8. Ruijter J, Ramakers C, Hoogaars W, Karlen Y, Bakker O, Van den Hoff M, Moorman A. 2009. Amplification efficiency: linking baseline and bias in the analysis of quantitative PCR data. Nucleic Acids Res 37:e45-e45.
9. Tuomi JM, Voorbraak F, Jones DL, Ruijter JM. 2010. Bias in the *Cq* value observed with hydrolysis probe based quantitative PCR can be corrected with the estimated PCR efficiency value. Methods 50:313-322.
10. Pfaffl MW. A 2001. new mathematical model for relative quantification in real-time RT–PCR. Nucleic Acids Res 29:e45-e45.
11. Febvay G, Delobel B, Rahbé, Y. 1988. Influence of the amino acid balance on the improvement of an artificial diet for a biotype of *Acyrthosiphon pisum* (Homoptera: Aphididae). Can J Zool 66:2449-2453.

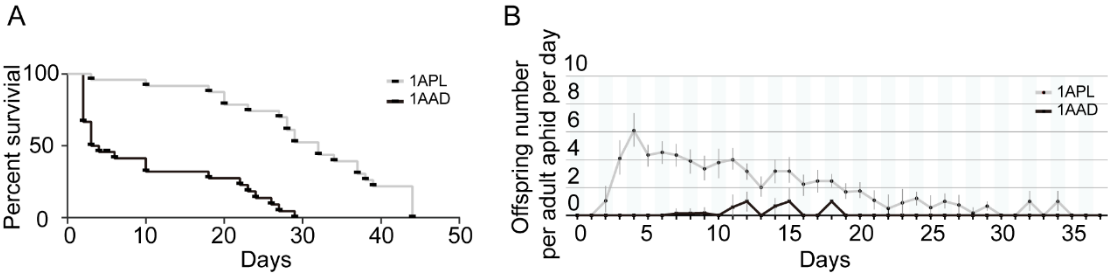

FIG S1 Aphids feeding on an artificial diet performed poorly relative to those feeding on plant materials. (A) Kaplan-Meier plot visualized the survival of 48 nymphs without primordia which were reared under 1APL (gray line) or 1AAD (black line) conditions. (B) Daily fecundity under 1APL (gray line) or 1AAD (black line) conditions. Means and standard errors are shown.

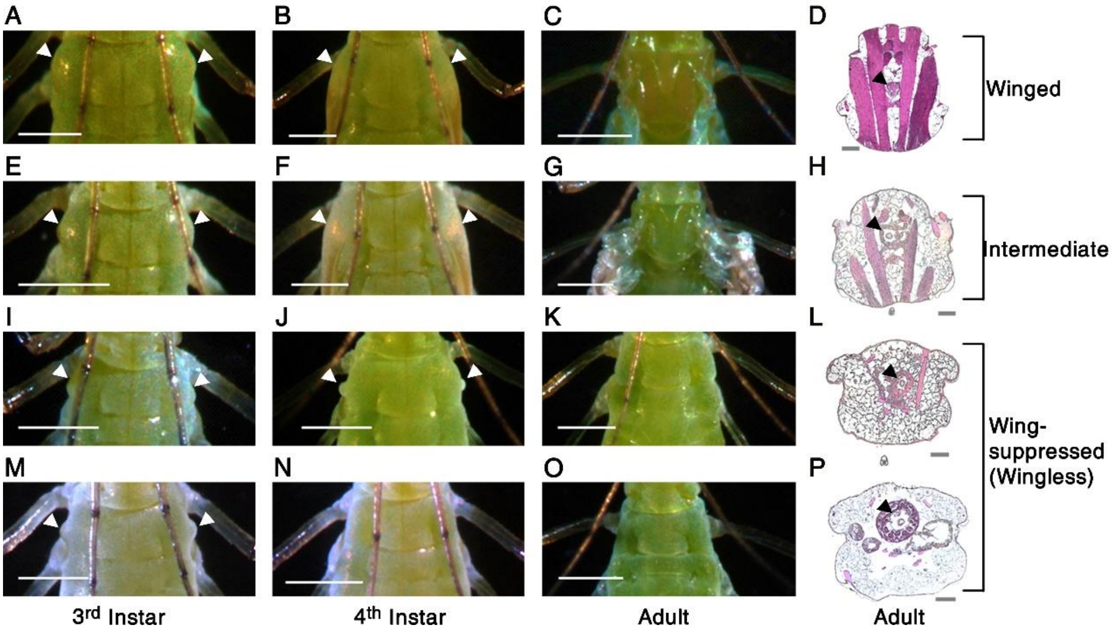

FIG S2 Wing structures were suppressed when apterization occurred in *A. pisum*. External thoracic morphology from the third instar to adult, and histological structure (wing muscles) of thoracic regions of different adults. Images in the same row show thoracic structures of the same aphid from the third instar to adult (A to C, E to G, I to K, and M to O), followed by a histological image of the adult thorax (D, H, L and P). White arrowheads indicate wing primordia, and the black arrowheads show the esophageal valve. The white and black scale bars are 500 μm and 100 μm, respectively.

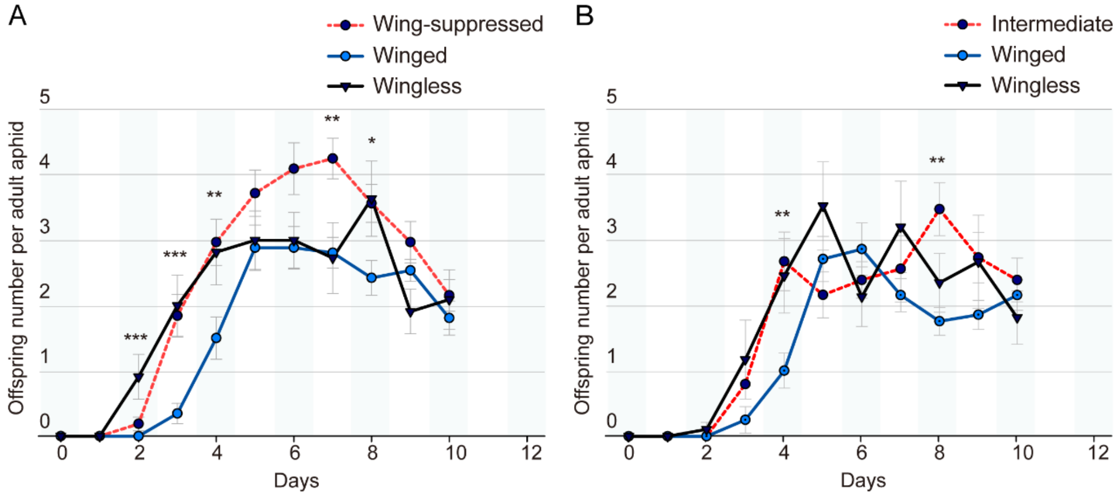

FIG S3 Daily fecundity of *Acyrthosiphon pisum* adults with different wing morphs. (A) Comparison of daily fecundity among the wing-suppressed (*n*= 32), winged (*n*= 26) and wingless (*n*= 11) adults. (B) Comparison of daily fecundity among the intermediate (*n*= 15), winged (*n*= 17) and wingless (*n*= 8) adults. Means and standard errors are shown. Means with *, ** and *** indicate differences at *P*< 0.05, < 0.01 and < 0.001, respectively (Brown-Forsythe test or Kruskal-Wallis test).

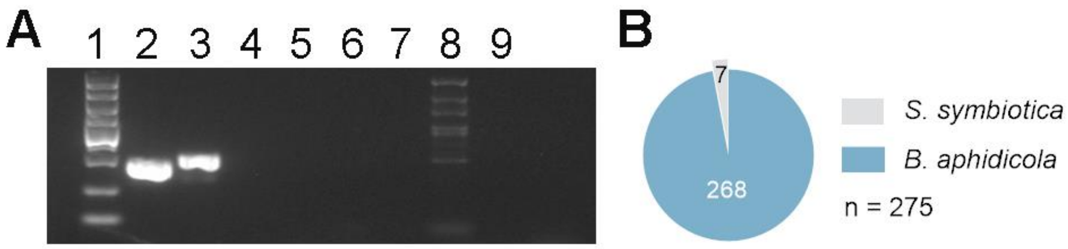

FIG S4 Only *B. aphidicola* and *S. symbiotica* were detected in the pea aphid. (A) Diagnostic PCR results of endosymbiont detection by specific primers on pea aphids. 1: Marker; 2: PCR result on *B. aphidicola*; 3: PCR result on *S. symbiotica*; 4: *Regiella insecticola*; 5: *Hamiltonella defense*; 6: *Richettsia* (16SrDNA); 7: *Richettsia* (citrate synthase); 8: *Spiroplasma* (16SrDNA); 9: *Spiroplasma* (dnaA). (B) Blast results of the 16s primer products.

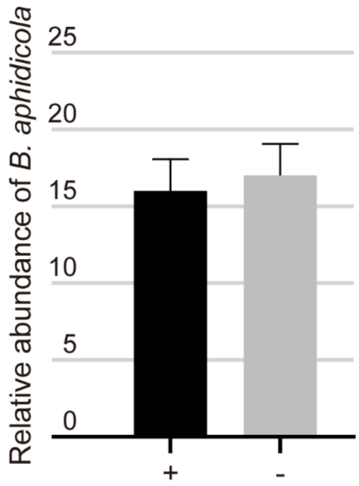

FIG S5 Impact of antibiotic treatment on the relative abundance of *B. aphidicola*. Plus sign (+) indicates the naturally-infected line and minus sign (-) refers to the cured line (antibiotic treatment).

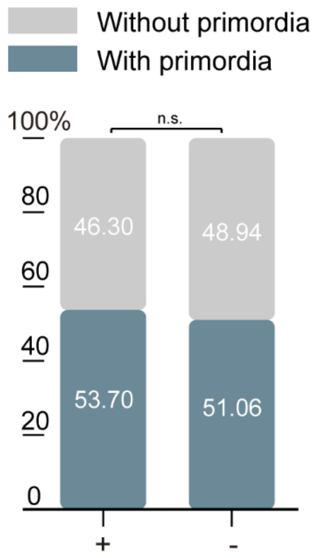

FIG S6 Percentages of different wing morphs of the third instar cured and naturally-infected aphids born under crowded condition and maintained under a poor-quality diet (Chi-square = 0.0702; *P* = 0.7910; contingency coefficient = 0.0264; *n* = 101).

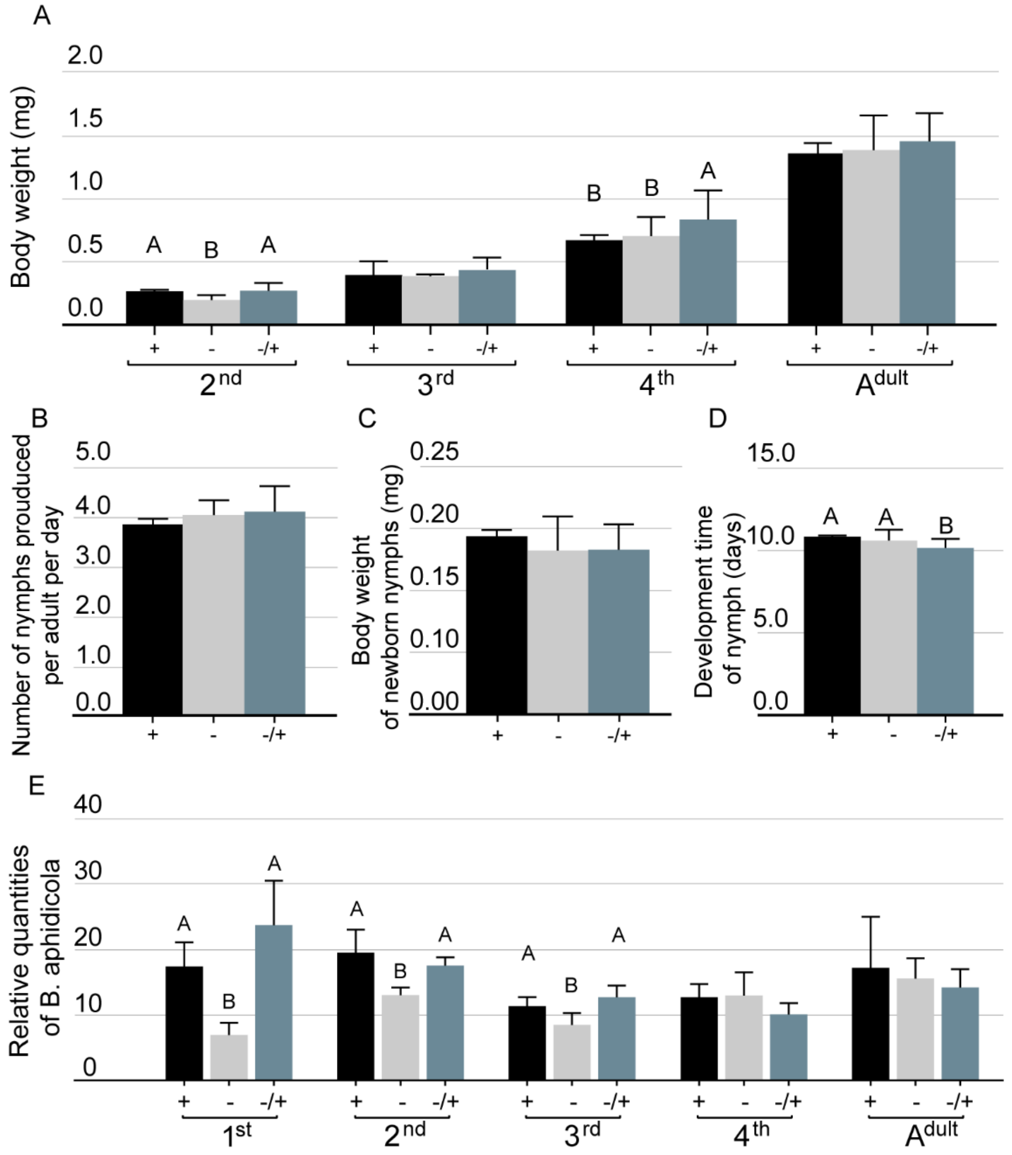

FIG S7 Effects of *S. symbiotica* on fitness of *A. pisum* and the primary symbiotica *Buchnera aphidicola*. (A) Body weight of wingless adults (+, *n*= 31; -, *n*= 35; -/+, *n*= 32). (B) Body weight of neonate nymphs. (C) Mean numbersof nymphs produced daily for 10 days (+, *n*= 32; -, *n*= 28; -/+, *n*= 32). (D) Development time of nymphs was measured by the time interval from birth to the final molt (+, *n*= 52; -, *n*= 36; -/+, *n*= 32). (E) The relative gene copy number of *B. aphidicola* decreased significantly in the first, second and third instar nymphs without *S. symbiotica* compared with naturally-infected and re-infected lines (*n*= 3). Columns sharing the same letters (A or B) are not different ($P > 0.05$; Tukey's pairwise comparison test). Means and standard errors are shown. Plus sign (+) indicates the naturally-infected line, minus sign (-) refers to the cured line, and minus and plus sign (-/+) shows the re-infected line.
